# Leanness and Low Plasma Leptin in GPR17 Knockout Mice Are Dependent on Strain and Associated With Increased Energy Intake That Is Not Suppressed by Exogenous Leptin

**DOI:** 10.3389/fendo.2021.698115

**Published:** 2021-09-27

**Authors:** Edward T. Wargent, Suhaib J. S. Ahmad, Qing Richard Lu, Evi Kostenis, Jonathan R. S. Arch, Claire J. Stocker

**Affiliations:** ^1^ Institute of Translational Medicine, University of Buckingham, Buckingham, United Kingdom; ^2^ Department of Surgery, Barts Cancer Institute, Queen Mary University of London, London, United Kingdom; ^3^ Division of Experimental Hematology and Cancer Biology, Department of Pediatrics, Brain Tumor Center, Cincinnati Children’s Hospital Medical Center, Cincinnati, OH, United States; ^4^ University of Bonn, Bonn, Germany; ^5^ Aston Medical School, Aston University, Birmingham, United Kingdom

**Keywords:** GPR 17 knockout mouse, genetic background, high fat diet, body composition, leptin resistance, energy expenditure, energy intake

## Abstract

Previous studies have shown that agonists of GPR17 stimulate, while antagonists inhibit feeding. However, whole body knockout of GPR17 in mice of the C57Bl/6 strain did not affect energy balance, whereas selective knockout in oligodendrocytes or pro-opiomelanocortin neurons provided protection from high fat diet-induced obesity and impaired glucose homeostasis. We reasoned that whole body knockout of GPR17 in mice of the 129 strain might elicit more marked effects because the 129 strain is more susceptible than the C57Bl/6 strain to increased sympathetic activity and less susceptible to high fat diet-induced obesity. Consistent with this hypothesis, compared to wild-type mice, and when fed on either a chow or a high fat diet, GPR17 -/- mice of the 129 strain displayed increased expression of uncoupling protein-1 in white adipose tissue, lower body weight and fat content, reduced plasma leptin, non-esterified fatty acids and triglycerides, and resistance to high fat diet-induced glucose intolerance. Not only energy expenditure, but also energy intake was raised. Administration of leptin did not suppress the increased food intake in GPR17 -/- mice of the 129 strain, whereas it did suppress food intake in GPR17 +/+ mice. The only difference between GPR17 +/- and GPR17 +/+ mice of the C57Bl/6 strain was that the body weight of the GPR17 -/- mice was lower than that of the GPR17 +/+ mice when the mice were fed on a standard chow diet. We propose that the absence of GPR17 raises sympathetic activity in mice of the 129 strain in response to a low plasma fuel supply, and that the consequent loss of body fat is partly mitigated by increased energy intake.

## Introduction

The worldwide prevalence of obesity has more than doubled since 1980. This rapid rise forecasts an increased burden from several diseases, most notably type 2 diabetes mellitus (1). The thrifty genotype hypothesis postulates that both obesity and type 2 diabetes mellitus are caused by a positive selection of genotypes for efficient metabolism and fat storage (2). Thus, identifying biochemical pathways to target these genes, that are amenable to pharmacological manipulation, holds promise for developing novel therapies for the treatment of metabolic disease.

Intracerebroventricularly-administered agonists of G protein-coupled receptor 17 (GPR17) stimulate and antagonists inhibit feeding, with the agonists promoting insulin resistance in response to high fat diet (3). GPR17 is a transcriptional target of FoxO1 (3, 4). FoxO1 expression is reduced by the anorexigenic hormones insulin and leptin. The effects of these hormones on feeding are inhibited by FoxO1 expression, increasing food intake (5), whereas mice that lacked FoxO1 in Agouti-related peptide neurones displayed reduced food intake, and were lean, with improved glucose homeostasis, and increased sensitivity to insulin and leptin (3). Leptin is released from adipocytes and signals the size of lipid stores and manifests mechanisms that affect metabolism, the metabolic syndrome and its cardiovascular complications (6, 7). Low levels stimulate feeding and reduce energy expenditure (8), but the ability of high levels to inhibit feeding is limited by the phenomenon of leptin resistance (9).

Mice that lack GPR17 might be predicted to have similar (beneficial) metabolic phenotypes to those that lack FoxO1. However, whole-body GPR17 null mice had similar food intake, body weight, metabolic rate and glucose homeostasis, in response to a high fat diet compared to wild-type mice (10). By contrast, selective knockout of GPR17 primarily in oligodendrocytes provided protection from high fat diet-induced obesity (11) and knocking out GPR17 in pro-opiomelanocortin neurons attenuated the metabolic effects of high fat diet on body weight and adiposity, most noticeably in female mice (12). Activation of pro-opiomelanocortin neurons increases sympathetic activity (13), so it is possible that increased sympathetic activity is the mechanism that links GPR17 knockout, in pro-opiomelanocortin neurons, with increased oxygen consumption. Sympathetic activation is a major influence on browning of adipose tissue (14, 15).

All the above cited studies on GPR17 null mice were conducted on mice, of the C57Bl/6 background strain. Wild-type C57Bl/6 mice have a lower capacity for browning of white adipose tissue (WAT) compared to mice of the 129 strain (16–20). Browning of WAT has been shown to protect against the metabolic effects of high fat diet (21–24), which may be why C57Bl/6 mice are more susceptible than 129 mice to the effects of high fat feeding (17–19, 25). We have therefore compared the effect of knocking out GPR17 on energy balance and glucose homeostasis in mice on C57Bl/6J and 129 backgrounds. We report beneficial changes in mice of the 129 but not the C57Bl/6J strain.

Interestingly, the knockout of GPR17 in mice on the 129 background not only makes them leaner with a higher energy expenditure than wildtype mice, but also their energy intake is raised. This condition is found in various other transgenic rodents, drug- or hormone-treated rodents and humans, and in response to exercise, but the mechanisms that stimulate energy intake have not been studied in detail. We show that increased energy intake in GPR17 null 129 mice cannot be due to low leptin levels because it is not suppressed by administration of exogenous leptin.

## Materials and Methods

Reagents were obtained from Sigma-Aldrich, Poole, UK, unless otherwise stated.

### Animals

Animal procedures were conducted in accordance with University of Buckingham project licence, under the UK Animals (Scientific Procedures Act (1986)) and as approved by the University’s Ethical Review Board. Three male and two female GPR17 -/- mice on a mixed 129 and C57Bl/6J background were donated by Professor Lu, Cincinnati Children’s Hospital, Cincinnati, USA. Genotypes were backcrossed for ten generations onto either a C57Bl/6NCrl or 129S2/SvPasCrl (Charles River, Germany). Twelve male and twelve female GPR17 +/+ and GPR17 -/- mice on 129 and C57Bl/6J background were fed a standard rodent diet (chow, n = 12; 10% fat, 70% carbohydrate and 20% protein by energy, Beekay Feed, B&K Universal Ltd., UK) or high fat diet (HFD, n = 12; 60% fat by energy value; Research Diets, New Brunswick, New Jersey; cat D12492) from age 5-6 weeks for 18 weeks. Mice were housed in pairs at 19 – 22°C with lights on at 08.00h, lights off at 20.00h, and were fed *ad libitum*.

### Food Consumption and Body Parameters

Mice were weighed at 23 to 24 weeks of age, when some had been on the HFD for 18 weeks. Body fat and lean content were measured using a Minispec LF90II Nuclear Magnetic Resonance (Bruker Corporation, Germany). Food consumption was then recorded for three consecutive days. From the evening of the day of the final measurement of food consumption, they were fasted overnight for 16 hours then re-fed. Food consumption was measured one hour after re-feeding. After two days, all mice were placed in clean cages without food 4 hours prior to lights out. Immediately prior to lights out, they were given an i.p. dose of either 5 ml.kg^-1^ saline or 20 mg.kg^+1^ leptin. All food consumption values are expressed as digestible energy content in kJ units. Energy expenditure was measured by open circuit indirect calorimetry, with mice in their home cages (26, 27). It was calculated from the flow of air exiting the respiratory chamber and the oxygen content of that air without removing CO_2_. This gives a more accurate estimate of energy expenditure than oxygen consumption provides (27–31).

### Oral Glucose Tolerance Test

After fasting for five hours, mice were dosed with glucose (3 g.kg^-1^, body weight PO by gavage). Blood samples were collected from the tail at -30, 0, + 30, +60 and +120 minutes, relative to glucose dosing. Blood glucose was measured using a glucose oxidase reagent kit (Gluc-PAP, GL2623; Randox, Crumlin, UK). Fasting plasma insulin (t -30 min) was measured by ELISA (Ultra-Sensitive Mouse Insulin ELISA kit, Catalog #: 90080; Crystal Chem, Downers Grove, IL, USA).

### Plasma Measurements

After fasting for five hours, blood samples were collected from the tail. Plasma leptin was measured by ELISA (Catalog # 90030; Chrystal Chem). Plasma NEFA (Catalog # FA115; Randox, Crumlin, UK) and triglycerides (Catalog # TR210; Randox, Crumlin, UK) were measured by colorimetric assay.

### Termination

At 24 weeks of age all mice were culled by concussion followed by cervical dislocation. Interscapular, perigenital and inguinal fat pads were dissected and weighed, the perigenital and inguinal weights presented being the average of the two pads for each. Inguinal fat pads were stored at -80°C for mRNA analysis.

### Inguinal Fat UCP-1 mRNA Analysis Using TaqMan RT-PCR

RNA analysis of UCP-1 in the inguinal fat was carried out using Micro Fluidic Cards (Applied Biosystems, Foster City, CA, USA) in accordance with the manufacturer’s protocol. The reactions were performed in duplicate for each sample using an Applied Biosystems 7900HT fast real-time PCR system. A standard curve was constructed for each gene using a serial dilution of cDNA from samples. The mean C_T_ values of the GPR17+/+ were then used to calculate the relative expression for each sample in the respective diet/sex cohort and data were normalized to *Ppia (cyclophilin)*. ATPAF1 was used as an internal control. Real time PCR (StepOne™, Applied Biosystems) was carried out using Assay on Demand pre-designed primer and probe sets (n = 12). Data were analyzed using the comparative ΔC_t_ method.

### Statistical Analysis

A priori power analysis was conducted using G*Power3 (32). Data are presented as mean ± SEM. The Student’s t-test was utilized for statistical comparisons, except for leptin-induced energy intakes, which were analyzed by 2-way ANOVA (factors: leptin and genotype). All data sets passed the Anderson-Darling test for normality of distribution (alpha of 0.05). The ROUT method was used to analyse data sets for outliers. No outliers were identified.

## Results

### GPR17 -/- 129 Mice Had Less Body Fat Than GPR17 +/+ Mice but There Was No Such Difference in C57Bl/6J Mice

**Figure 1:**
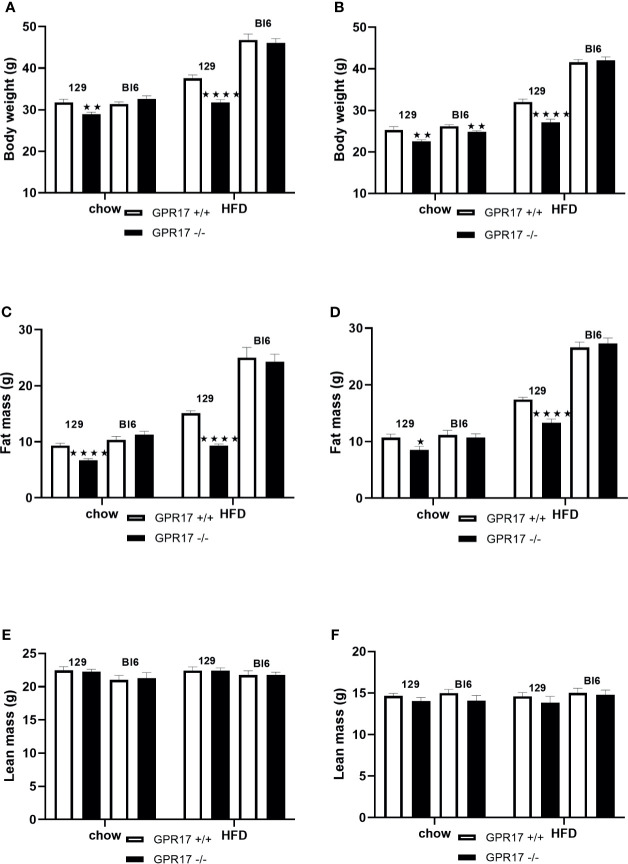
Body weight [**(A)** males, **(B)** females], fat mass **(C)** males, **(D)** females] and lean mass [**(E)** males, **(F)** females] in GPR17 +/+ (white bars) and GPR17 -/- (black bars) 129 and Bl6 mice fed standard chow or high fat diet for 18 weeks from age 6 weeks. N = 12 per group). ★*P*<0.05; ★★*P*<0.01; ★★★★*P*<0.0001. Values are expressed as means ± S.E.M.

Both male (*p* < 0.01) and female (*p* < 0.01) GPR17 -/- 129 mice had a lower whole-body weight’than GPR17 +/+ mice (**Figures 1A, B**). The difference remained when the mice were fed HFD for 18 weeks (*p* < 0.0001 for both males and females). This was due to fat mass being lower in the GPR17 -/- mice (males: *p* < 0.0001 on both chow and HFD; **Figure 1C**; females: *p* < 0.05 on chow, *p* < 0.0001 on HFD; **Figure 1D**), there being no difference in lean mass between GPR17 +/+ and +/- mice of either sex (**Figures 1E, F**).

Female but not male GPR17 -/- C57Bl/6J mice fed on chow had a lower whole-body weight than GPR17 +/+ (*p* < 0.01), but there were no differences in fat or lean mass between GPR17 +/+ and -/- genotypes in C57Bl/6J mice of either sex (**Figure 1**).

### GPR17 -/- 129, but Not C57Bl/6J, Mice on a High Fat Diet Had Smaller Adipose Tissue Depots

**Figure 2:**
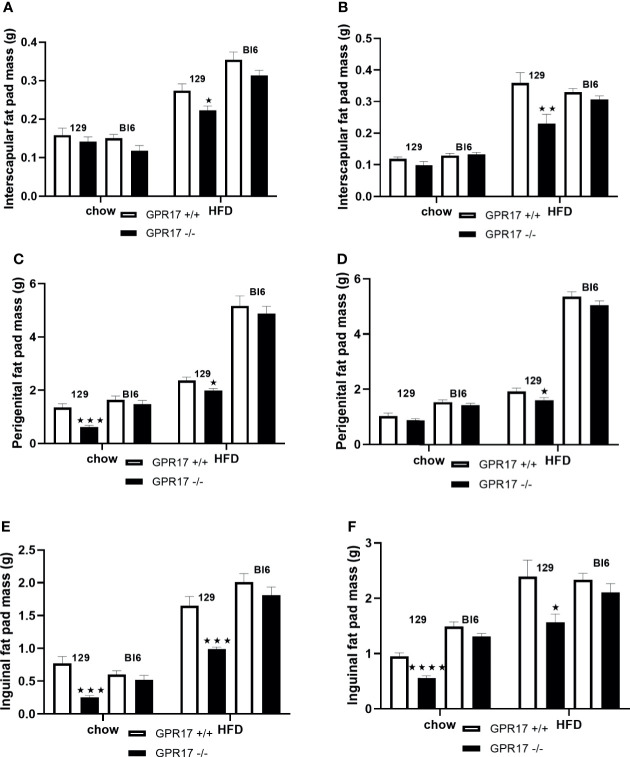
Fat pad weights in GPR17 +/+ (white bars) and GPR17 -/- (black bars) 129 and Bl6 mice fed standard chow or high fat diet for 18 weeks from age 6 weeks. Interscapular fat pad [**(A)** males, **(B)** females]; perigenital fat pad [**(C)** males, **(D)** females]; inguinal fat pad [**(E)** males, **(F)** females]. (N = 12 per group). ★*P*<0.05; ★★*P*<0.01; ★★★*P*<0.001; ★★★★*P*<0.0001. Values are expressed as means ± S.E.M.

On a standard chow diet, male mice had smaller perigenital (*p* < 0.001, **Figure 2C**) and inguinal (*p* < 0.001, **Figure 2E**), but not interscapular fat pads (**Figure 2A**). Female GPR17 -/- 129 mice on standard chow also had smaller inguinal (*p* < 0.0001, **Figure 2F**) but not interscapular or perigenital fat pads (**Figures 2B, D**). Both male and female GPR17 -/- 129 mice fed the HFD had significantly smaller interscapular, perigenital and inguinal fat pad depots than GPR17 +/+ mice (**Figures 2A–F**).

There was no statistically significant difference in fat pad weight between the genotypes on a C57Bl/6 background of either sex (**Figure 2**).

### GPR17 -/- 129, but Not GPR17 -/- C57Bl/6J, Mice Exhibited Increased Energy Intake and Energy Expenditure

**Figure 3:**
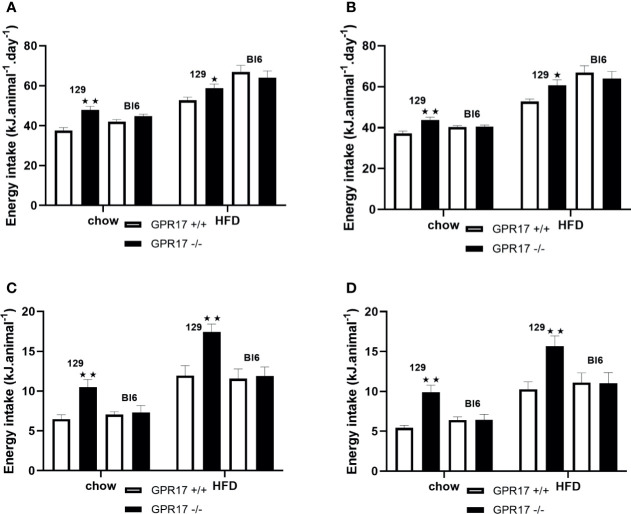
Energy intake in GPR17 +/+ (white bars) and GPR17 -/- (black bars) 129 and Bl6 mice fed standard chow or high fat diet for 18 weeks from age 6 weeks. Three-day average energy intake [**(A)** males, **(B)** females]; energy intake over the first hour of re-feeding after a 16 hour overnight fast [**(C)** males, **(D)** females]; energy intake over the first hour of dark period following a saline or leptin i.p. dose. (N = 6 per group). ★*P*<0.05; ★★*P*<0.01. Values are expressed as means ± S.E.M.

Despite being lighter and leaner, both male (*p* < 0.01, **Figure 3A**) and female (*p* < 0.01, **Figure 3B**) GPR17 -/- 129 mice had increased energy intake at 24 weeks of age. This was also the case when the mice were fed a HFD (*p* < 0.05). Food consumption was also higher in GPR17 +/- than GPR17 +/+ 129 mice after a 16 hour overnight fast, (*p* < 0.01 for males, **Figure 3C**); *p* < 0.01 for females, **Figure 3D**). The increase in feeding following a fast also occurred with the HFD (*p* < 0.01 for both males and females). There were no effects of genotype on energy intake in C57Bl/6J mice (**Figure 3**).

**Figure 4:**
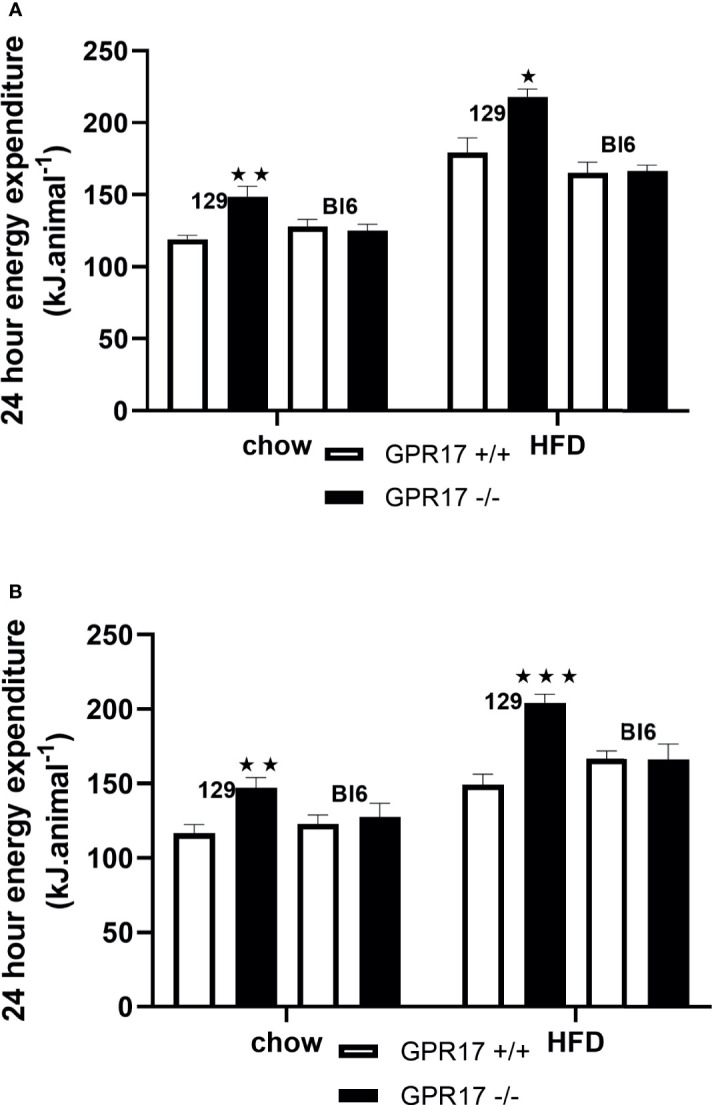
24-hour energy expenditure [**(A)** males, **(B)** females] in GPR17 +/+ (white bars) and GPR17 -/- (black bars) 129 and Bl6 mice fed standard chow or high fat diet for 18 weeks from age 6 weeks. N = 6 per group). ★*P*<0.05; ★★*P*<0.01; ★★★*P*<0.001. Values are expressed as means ± S.E.M.

To allow comparison with the energy intake data and because energy expenditure relative to body weight is affected by body composition (25), energy expenditure is expressed per animal. Both chow-fed male (*p* < 0.01, **Figure 4A**) and female (*p* < 0.01, **Figure 4B**) GPR17 -/- mice had increased twenty-four-hour energy expenditure at 24 weeks of age. Energy expenditure was also increased in GPR17 -/- mice fed on a HFD (*p* < 0.05 for males and *p* < 0.001 for females). No differences were found between GPR17 +/+ and -/- genotypes in C57Bl/6J mice (**Figure 4**).

### GPR17 -/- 129 Mice, but Not C57Bl/6J Mice, Were Protected Against HFD-Induced Glucose Intolerance and Insulin Resistance

**Figure 5:**
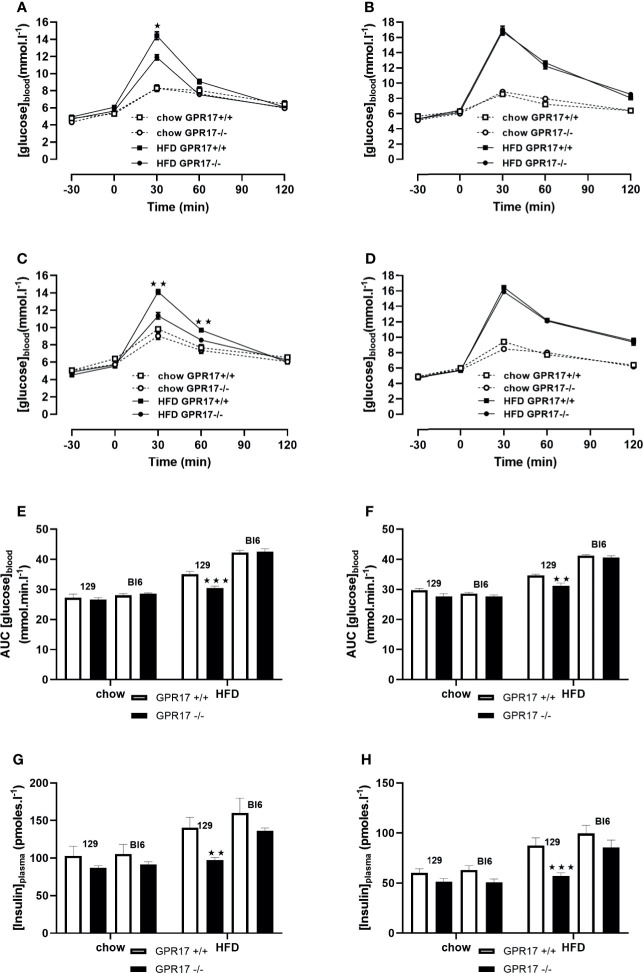
Oral Glucose tolerance test: blood glucose concentration curves [**(A)** 129 males, **(B)** C57Bl6 males, **(C)** 129 females, **(D)** C57Bl6 females]; area under the blood glucose concentration curve [**(E)** males, **(F)** females]; fasting plasma insulin concentration [**(G)** males, **(H)** females] in GPR17 +/+ (white bars) and GPR17 -/- (black bars) 129 mice fed standard chow or high fat diet for 18 weeks from age 6 weeks. N = 12 per group). ★*P*<0.05; ★★*P*<0.01; ★★★*P*<0.001. Values are expressed as means ± S.E.M.

18 weeks of HFD induced glucose intolerance in GPR17 +/+ mice. This effect was significantly attenuated in the GPR17 -/- 129 mice (*p* < 0.001 in males, **Figures 5A, B, E**; *p* < 0.01 in females, **Figures 5C, D, F**). Concordantly, the elevated fasting plasma insulin induced by HFD was significantly reduced in in the GPR17 -/- 129 mice (*p* < 0.01 in males, **Figure 5G**; *p* < 0.001 in females, **Figure 5H**). There was no difference between chow-fed C57Bl/6J and 129 mice in fasting blood glucose concentration in both males and females. No differences in either glucose tolerance or plasma insulin were found between GPR17 +/+ and -/- genotypes in C57Bl/6J mice (**Figure 5**).

### GPR17 -/- Mice Had Elevated UCP-1 Gene Expression in Inguinal Fat

**Figure 6:**
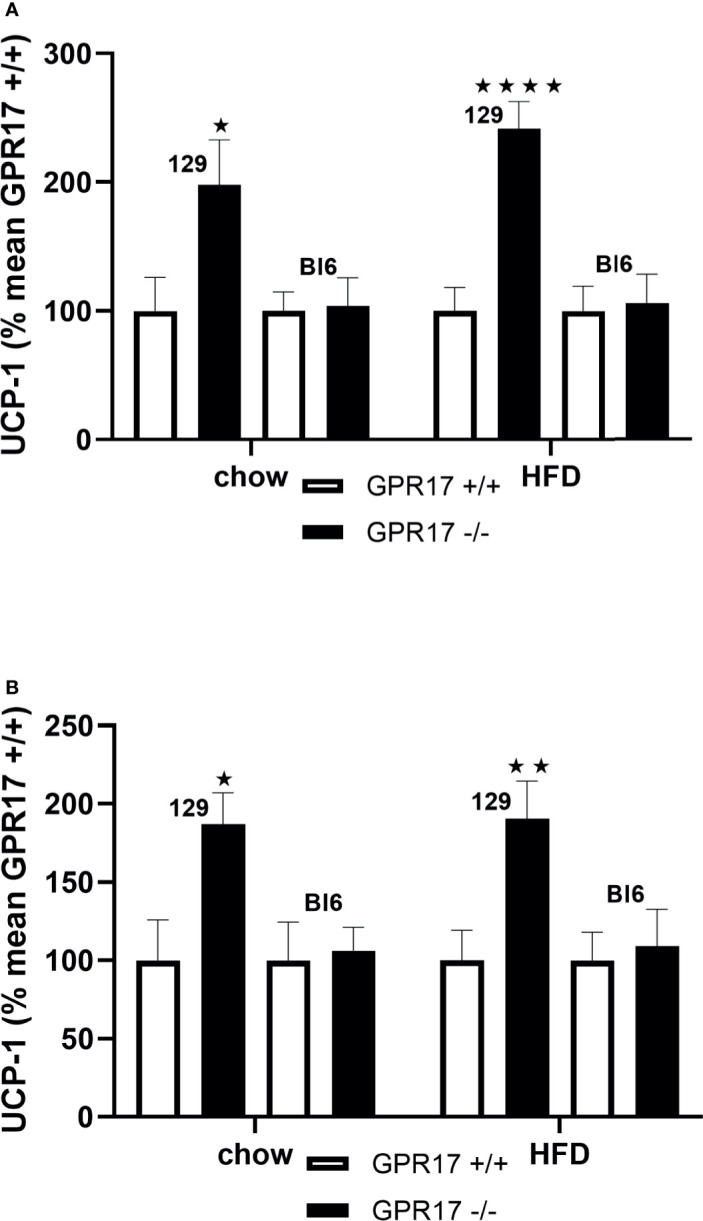
Inguinal fat pad UCP-1 mRNA levels [**(A)** males, **(B)** females] in GPR17 +/+ (white bars) and GPR17 -/- (black bars) 129 and Bl6 mice fed standard chow or high fat diet for 18 weeks from age 6 weeks. N = 6 per group). ★*P*<0.05; ★★*P*<0.01, ★★★★*P*<0.01. Values are expressed as means ± S.E.M.

Both chow-fed male (*p* < 0.05, **Figure 6A**) and female (*p* < 0.05, **Figure 6B**) GPR17+/- mice on the 129 background had increased inguinal fat UCP-1 gene expression. Inguinal fat UCP-1 was also increased in GPR17 -/- 129 mice fed a HFD (*p* < 0.0001 for males and *p* < 0.01 for females). No differences were found in UCP-1 gene expression between GPR17 +/+ and -/- genotypes in C57Bl/6J mice (**Figure 6**).

### Plasma Lipids and Leptin Levels Were Lower in GPR17 -/- 129 mice, but Not C57Bl/6 Mice

**Figure 7:**
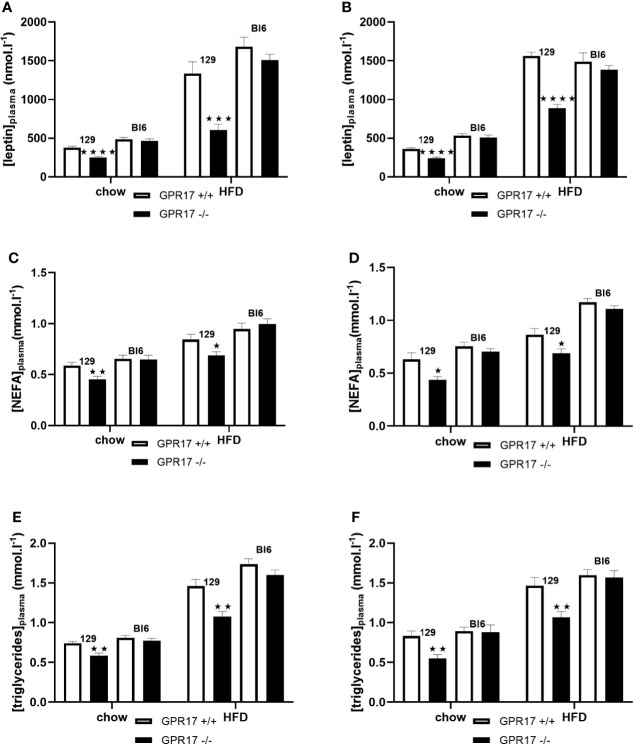
Plasma leptin and lipids in GPR17 +/+ (white bars) and GPR17 -/- (black bars) 129 and Bl6 mice fed standard chow or high fat diet for 18 weeks from age 6 weeks. Leptin [**(A)** males, **(B)** females]; NEFA [**(C)** males, **(D)** females]; triglycerides [**(E)** males, **(F)** females]. (N = 12 per group). ★*P*<0.05; ★★*P*<0.01, ★★★*P*<0.001; ★★★★*P*<0.0001. Values are expressed as means ± S.E.M.

When fed on the chow diet, both male (*p* < 0.0001) and female (*p* < 0.0001) GPR17 -/- 129 mice had lower plasma leptin concentrations compared to GPR17 +/+ mice (**Figures 7A, B**). This effect of genotype was also present when the mice were fed HFD for 18 weeks (*p* < 0.001 for males and *p* < 0.0001 for females). Plasma non-esterified fatty acids (NEFA) concentration was also lower in the GPR17 -/- mice (males: *p* < 0.01 on chow and *p* < 0.05 on HFD, **Figure 7C**; females: p < 0.05 on both chow and HFD, **Figure 7D**). Similarly, plasma triglycerides concentration was lower in GPR17 -/- mice (males: *p* < 0.01 on chow and HFD, **Figure 7E**; females: p < 0.01 on both chow and HFD, **Figure 7F**).

No effects of genotype on plasma leptin, NEFA or triglycerides were found in C57Bl/6J mice (**Figure 7**).

### Leptin Reduced Energy Intake in GPR17 +/+ but Not -/- 129 Mice

**Figure 8:**
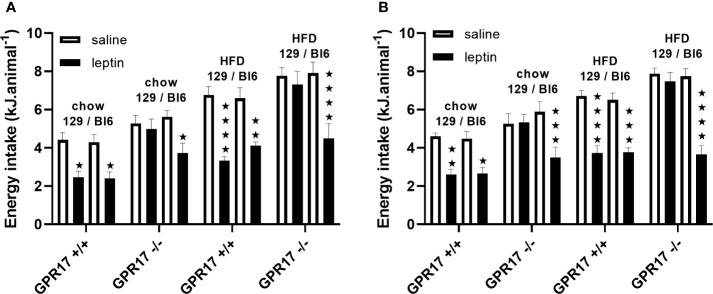
Energy intake over the first hour of dark period following a saline or leptin i.p. dose in 129 and Bl6 mice [**(A)** males, **(B)** females]. (N = 6 per group). ★*P*<0.05; ★★*P*<0.01; ★★★*P*<0.001; ★★★★*P*<0.0001. Values are expressed as means ± S.E.M.

To investigate a possible role of low leptin levels in increased energy intake in GPR17 -/- 129 mice, food intake was measured following administration of leptin. Contrary to this hypothesis, leptin did not reduce food intake in male or female GPR17 -/- 129 mice on either diet, whereas it did reduce food intake in GPR17 +/+ 129 mice (males: *p* < 0.05 on chow, < 0.0001 on HFD; females: *p* < 0.01 on chow, < 0.0001 on HFD; **Figures 8A, B**).

By contrast, leptin reduced food inake in both GPR17 +/+ and -/- genotypes in C57Bl/6J mice, there being no difference between the GPR17 genotypes (**Figures 8A, B**).

## Discussion

We have compared the effect of knocking out GPR17 on energy balance and glucose handling in mice on a C57Bl/6J background, which are considered susceptible to high fat feeding, and in mice on a 129 background, which are considered to be resistant (23). Both male and female mice, and mice fed on a chow diet or on a high fat diet for 18 weeks from the age of six weeks were compared. Compared to wild type mice and irrespective of their sex or diet, GPR17 -/- mice on a 129 background were lighter due to a decreased body fat content and had increased energy expenditure (the difference in energy expenditure would be more marked if the results were expressed relative to body weight because the GPR17 -/- mice are lighter than the GPR17 +/+ mice). They were also less glucose intolerant than the wild type mice after they had been fed on the high fat diet. Other than a reduction in body weight when the female mice were fed on chow, none of these differences between GPR17 -/- and wild type mice were found in mice on a C57Bl/6J background. Again, irrespective of their sex or diet, GPR17 -/- mice on a 129 background, but not mice on the C57Bl/6J background, had increased inguinal fat UCP-1 gene expression compared to wildtype mice.

A possible explanation that links all these findings is that GPR17 -/- mice on a 129 but not on a C57Bl/6J background had increased sympathetic activity. Thus, sympathetic activation is the major influence on browning of adipose tissue (14, 15); wild-type C57Bl/6 mice have a lower capacity for browning of white adipose tissue than mice of the 129 strain (17–19, 23); and sympathomimetic agents, such as β3-adrenoceptor agonists, display all the beneficial effects on energy balance and glucose homeostasis that we report here (33, 34). We chose to measure the level of UCP-1 mRNA in white adipose tissue because it is a well-established marker of sympathetic activity. Consistent with our hypothesis, this was increased in GPR17 -/- mice on a 129 but not on a C57Bl/6J background. Studies that directly measure sympathetic activity are needed to confirm our hypothesis. In addition, studies in primary cultures of preadipocytes from mice of both strains and genotypes would show whether differences in UCP-1 expression in response to a sympathomimetic agent, such as isoproterenol or a β3-adrenoceptor agonist, are cell-autonomous and independent of sympathetic activity *in vivo*. A limitation of the present study is a lack of scope to provide a more complete characterization of the browning process. Other studies have been conducted with pharmacological agents that mimic the activity of the SNS (29, 33). Those studies were often conducted in C57Bl rather than 129 mice, but it is the C57Bl mice with their lower baseline sympathetic tone where one might expect to see the greater effect. A comparison of the effects of a β3-adrenoceptor agonist (for example) in diet-induced obese C57Bl and 129 mice would be interesting, but this would merit a paper of its own. Studies that directly measure sympathetic activity are needed to support our hypothesis. If there are any such differences, a possible role for glucagon-like peptide-1, which affects both energy intake and energy expenditure, should be considered (35). Similarly, whilst not being within the scope of this report, investigation into the possible role of other hormones that affect energy intake and metabolism, such as GLP-1 and adiponectin, ought to be conducted. Quantification of adipocyte size and number to determine whether the fat depots are smaller due to cell size or number, along with the adipocytes' lipid metabolism mechanisms, would also be valuable, as these parameters have been associated with murine susceptibility/resistance to high-fat diet (36).

Other workers have also found no effect on body weight, food intake or energy expenditure of whole-body knock out of GPR17 in C57Bl/6 mice (10), though knocking out GPR17 in C57Bl/6 mice in oligodendrocytes (11) or pro-opiomelanocortin neurones (12) has been reported to affect energy balance and glucose homeostasis. Activation of pro-opiomelanocortin neurons increases sympathetic activity (13), consistent with our suggestion that the effects on energy balance, glucose homeostasis and UCP-1 expression of knocking out GPR17 that we report here are mediated by increased sympathetic activity. As well as the type of knockout, the temperature at which the mice were housed and the numbers of mice per cage could play a role in accounting for different findings in GPR17 -/- mice on the C57Bl/6 background (37–40). The mice were housed in groups of four to five (10) or three to five (12) in previous studies, and at a temperature range of 23 ± 1°C. However, differences in the housing temperature and the number of mice per cage cannot account for our different findings in GPR17 -/- mice on the C57Bl/6 and 129 backgrounds because mice of both strains were held in pairs and at a temperature of 19 to 22°C.

The finding that increased energy intake in GPR17 -/- 129 mice is accompanied by increased energy expenditure is far from unique. For example, acetyl-CoA carboxylase-2 knockout and 11-hydroxysteroid dehydrogenase-1 knockout mice are leaner than wild-type mice, despite having a higher energy intake (29). Hyperthyroidism is associated with increased energy intake, and in some studies in rodents, the thermogenic effect on body weight of administration of thyroid hormones has been offset by increased energy intake (41–43). Prolonged strenuous physical activity also increases food intake (44).

Another example of increased energy expenditure being accompanied with increased energy intake is when β_3_- adrenoceptor agonists are administered daily, by the oral route, to lean animals (45, 46). Together with a lower thermogenic response in lean compared to obese mice, this adaptation prevents the lean animals from being totally depleted of fat, their having less fat in total than the amount that is depleted in obese animals. For example, in one study, treatment for four weeks with the thermogenic β3-adrenoceptor agonist BRL26830A resulted in a body lipid content in genetically obese (ob/ob) mice that was 7 g lower than in control mice. By contrast, body lipid content in control lean mice was only 4.7 g, so it was not possible for body lipid content in BRL26830A-treated mice to be 7 g lower than 4.7 g. In fact, it was 1.2 g lower (45). Suggested mechanisms for increased energy intake in response to β_3_-adrenoceptor agonists in lean animals have been low plasma non-esterified fatty acid (NEFA) and low leptin levels in the lean animals (47). NEFA and leptin levels were, indeed, low compared to wild-type mice in the GPR17-/- mice on the 129 but not the C57Bl/6 background. Plasma triglyceride levels, which have also been proposed to regulate feeding (48) were similarly low. The hypothesis that low leptin levels stimulate energy intake to prevent excessive depletion of lipid stores can, however, be excluded in the case of GPR17-/- mice on the 129 background. First, the relative increase in energy intake in GPR17-/- compared to GPR17+/+ mice was similar in chow and HFD-fed mice. The low leptin hypothesis might predict a differential diet effect on energy consumption considering the plasma leptin levels in mice fed a HFD relative to chow diet. More conclusively, rather than being hypersensitive to leptin, increased energy intake in the GPR17-/- mice on the 129 background was resistant to suppression by administration of leptin, whereas leptin suppressed energy intake in the wild-type mice (**Figure 8**). Thus, the mechanism that accounts for increased energy intake overrides any effect of leptin. The role of low NEFA and triglyceride levels in stimulating energy intake (48, 49) in GPR17-/- mice merits further investigation. Low plasma levels of these fuels due to increased energy expenditure may stimulate energy intake by lowering hypothalamic malonyl CoA levels (50).

In summary, compared to wild-type mice, GPR17 -/- mice of the 129 strain displayed increased expression of UCP-1 in white adipose tissue, lower body weight and fat content, and resistance to HFD-induced glucose intolerance. Both energy expenditure and energy intake were raised. Administration of leptin did not suppress food intake in GPR17 -/- mice of the 129 strain, whereas it did suppress food intake in GPR17 +/+ mice. The only difference between GPR17 -/- and GPR17 +/+ mice of the C57Bl/6 strain was that the body weight of the female GPR17 -/- mice was lower than that of the GPR17 +/+ mice when the mice were fed on a standard chow diet. We suggest that the differences between strains are due to mice of the 129 strain tending to have a higher sympathetic activity than mice of the C57Bl/6 strain. Raised energy intake was not a consequence of low leptin levels and may have been due to a lower plasma level of a fuel, such as triglyceride or NEFA.

## Data Availability Statement

The original contributions presented in the study are included in the article/**Supplementary Material**. Further inquiries can be directed to the corresponding author.

## Ethics Statement

The animal study was reviewed and approved by University of Buckingham Animal Welfare and Ethical Review Board.

## Author Contributions

EW co-designed, co-ordinated and performed the *in vivo* study, and also generated all physiological data and co-wrote the initial drafts of the manuscript. SA generated the molecular biology UCP-1 tissue expression data. QL co-designed the study and generated the transgenic mice. EK co-originated the study and co-wrote the manuscript. JA wrote a large proportion of the manuscript. CS principal investigator: co-originated, co-designed and co-ordinated the institutional groups. All authors contributed to the article and approved the submitted version.

## Funding

The study was funded in its entirety internally by the Institute of Translational Medicine, University of Buckingham, Buckingham, MK18 1EG, UK

## Conflict of Interest

The authors declare that the research was conducted in the absence of any commercial or financial relationships that could be construed as a potential conflict of interest.

## Publisher’s Note

All claims expressed in this article are solely those of the authors and do not necessarily represent those of their affiliated organizations, or those of the publisher, the editors and the reviewers. Any product that may be evaluated in this article, or claim that may be made by its manufacturer, is not guaranteed or endorsed by the publisher.
